# A framework for computing angle of progression from transperineal ultrasound images for evaluating fetal head descent using a novel double branch network

**DOI:** 10.3389/fphys.2022.940150

**Published:** 2022-12-02

**Authors:** Jieyun Bai, Zhanhang Sun, Sheng Yu, Yaosheng Lu, Shun Long, Huijin Wang, Ruiyu Qiu, Zhanhong Ou, Minghong Zhou, Dengjiang Zhi, Mengqiang Zhou, Xiaosong Jiang, Gaowen Chen

**Affiliations:** ^1^ College of Information Science and Technology, Jinan University, Guangzhou, China; ^2^ Guangdong Provincial Key Laboratory of Traditional Chinese Medicine Information Technology, Jinan University, Guangzhou, China; ^3^ Obstetrics and Gynecology Center, Zhujiang Hospital, Southern Medical University, Guangzhou, China

**Keywords:** angle of progression, transperineal ultrasound image, pubic symphysis, fetal head, image segmentation, deep learning

## Abstract

**Background:** Accurate assessment of fetal descent by monitoring the fetal head (FH) station remains a clinical challenge in guiding obstetric management. Angle of progression (AoP) has been suggested to be a reliable and reproducible parameter for the assessment of FH descent.

**Methods:** A novel framework, including image segmentation, target fitting and AoP calculation, is proposed for evaluating fetal descent. For image segmentation, this study presents a novel double branch segmentation network (DBSN), which consists of two parts: an encoding part receives image input, and a decoding part composed of deformable convolutional blocks and ordinary convolutional blocks. The decoding part includes the lower and upper branches, and the feature map of the lower branch is used as the input of the upper branch to assist the upper branch in decoding after being constrained by the attention gate (AG). Given an original transperineal ultrasound (TPU) image, areas of the pubic symphysis (PS) and FH are firstly segmented using the proposed DBSN, the ellipse contours of segmented regions are secondly fitted with the least square method, and three endpoints are finally determined for calculating AoP.

**Results:** Our private dataset with 313 transperineal ultrasound (TPU) images was used for model evaluation with 5-fold cross-validation. The proposed method achieves the highest Dice coefficient (93.4%), the smallest Average Surface Distance (6.268 pixels) and the lowest AoP difference (5.993°) by comparing four state-of-the-art methods. Similar results (Dice coefficient: 91.7%, Average Surface Distance: 7.729 pixels: AoP difference: 5.110°) were obtained on a public dataset with >3,700 TPU images for evaluating its generalization performance.

**Conclusion:** The proposed framework may be used for the automatic measurement of AoP with high accuracy and generalization performance. However, its clinical availability needs to be further evaluated.

## Highlights

- A framework for evaluating fetal head descent is proposed.
- The framework includes image segmentation, target fitting and measurement of the angle of progression (AoP).
- A novel double branch network (DBSN) is proposed based on a U-shaped architecture.
- Experimental results show that the proposed DBSN outperforms existing networks in computing AoP.

## 1 Introduction

**FIGURE. 1:**
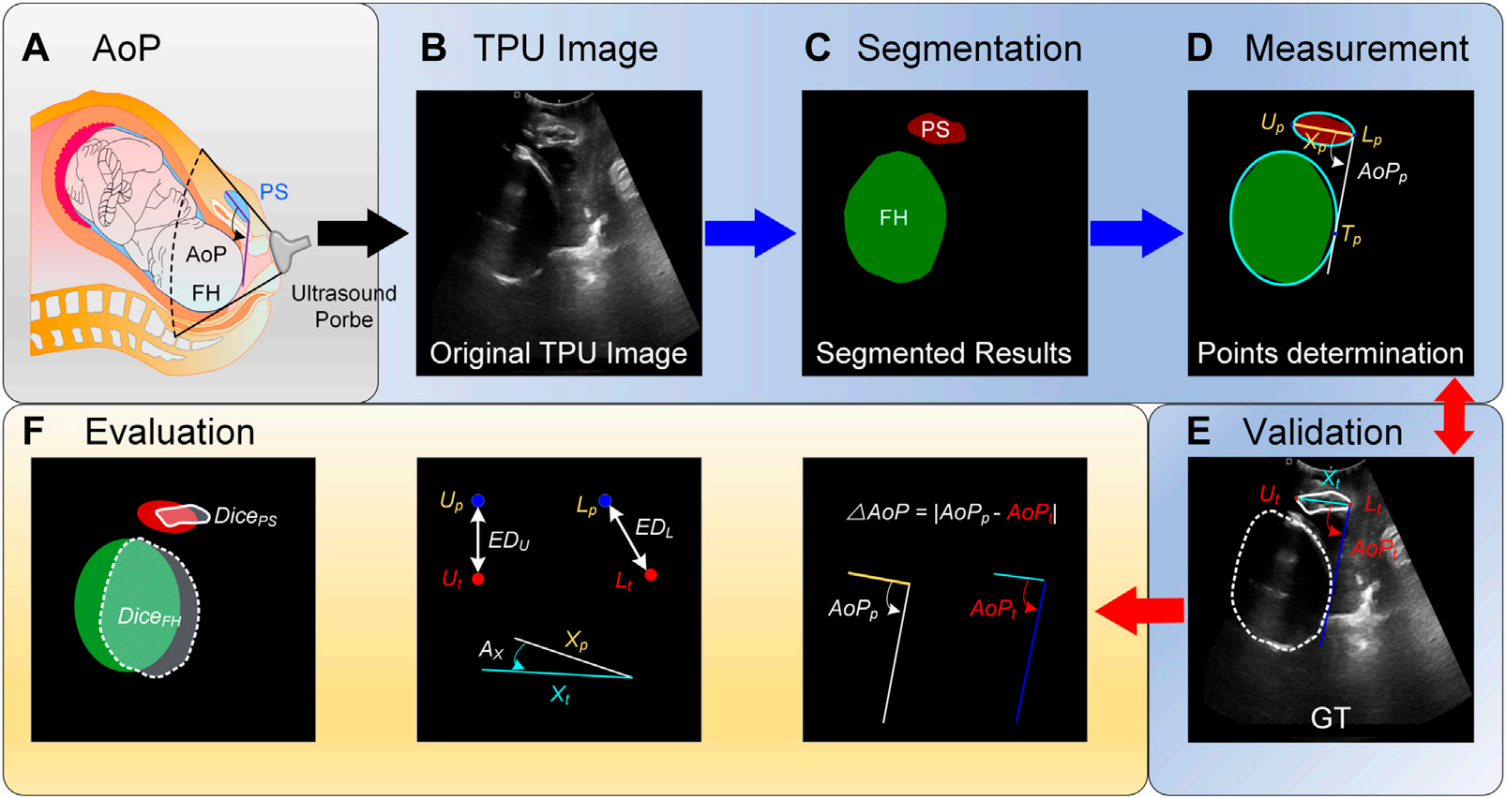
Schematic diagram of the angle of progression (AoP) that is measured with our proposed method. **(A)** AoP is defined as the angle between a line through the long axis of the pubic symphysis (PS) and a second line from the inferior end of the symphysis pubis tangentially to the contour of the fetal head (FH). **(B)** Images are firstly obtained with the transperineal ultrasound (TPU) probe. **(C)** Areas of PS and FH are segmented with the proposed method. **(D)** Segmented areas are fitted with elliptic equations and thereby three key points for computing AoP are determined. Compared with ground truths (GTs) **(E)**, the proposed method is evaluated with several metrics **(F)**.

The high risk of maternal and perinatal morbidity is associated with longer labor duration due to the slow progression of fetal descent (Fitzpatrick et al., 2001), but accurate assessment of fetal descent by monitoring the fetal head (FH) station remains a clinical challenge in guiding obstetric management (Simkin, 2010). Based on clinical findings (Zhang et al., 2010; Segel et al., 2012; Hamilton et al., 2016), the transvaginal digital examination is the most commonly used clinical estimation method of fetal station (Oboro et al., 2005; Boyle et al., 2013; Cohen et al., 2017). However, this traditional approach is very subjective, often difficult, and unreliable (Sherer et al., 2002; Dupuis et al., 2005). The need of an objective diagnosis found its solution in the use of transperineal ultrasound (TPU) able to assess FH station by measuring the angle of progression (AoP) that is the extension the FH goes through in its descent (Figure 1). The AoP first described in 2009 (Barbera et al., 2009; Kalache et al., 2009) is defined as the angle between the long axis of the pubic symphysis (PS) and a line from the lower endpoint of PS drawn tangential to the FH contour (Ghi et al., 2009; Dückelmann et al., 2010; Youssef et al., 2013). Several studies have suggested that AoP is an objective, accurate, reliable and reproducible parameter for the assessment of FH descent to provide the best diagnosis that will support the clinician in his/her daily decision (Montaguti et al., 2018; Dall’asta et al., 2019; Brunelli et al., 2021; Youssef et al., 2021).

The AoP computed from the two-dimensional TPU images is based on the areas of PS and FH (Burgos-Artizzu et al., 2020). Although the pipeline is easy to handle, the following disadvantages still exist. On the one hand, high variability between and within operators, especially for novices, results in subjective inaccuracy. On the other hand, it is time-consuming for real-time measurement when AoP changes need to be monitored during the second stage of labor. For FH descent monitoring, manual measurement of AoPs and examination bring huge difficulty and decrease the efficiency. Developing an automatic AoP measurement algorithm would be a possible solution to alleviate these problems. Conversano et al. (2017) first proposed a novel method, which used the combination of morphological filters and pattern recognition methods to identify PS-FH and calculate AoPs from videos. For each video, the ultrasound standard plane was first selected based on geometrical features and the gray level. Bone structures were secondly determined for the first image or the subsequent images. PS-FH for the first image was segmented based on the pixel position and gray value intensities, whereas PS-FH for the subsequent images was detected based on morphological images from the first acquisition session or pattern tracking methods for images from subsequent acquisition sessions. Thirdly, segmented results were manually selected to co-registrant coordinates for PS-FH. Finally, AoP was measured. Unlike Conversano et al. (2017), Zhou et al. and Lu et al. proposed a deep learning-based framework for segmenting the region of PS-FH and locating the landmark of PS endpoints from ultrasound standard planes. The central axis of PS was then obtained with the localization of the landmarks, while the tangent of FH was computed. Finally, AoP was measured from the central axis and the tangent point (Zhou et al., 2020; Lu et al., 2022a).

It is worth noting that the error of AoP measurement greatly depends on the size and shape of the segmented PS-FH that are easily affected by TPU image quality. On the one hand, it is the small object semantic segmentation for PS relative to FH and there are two endpoints in segmented PS for computing AoP. Therefore, the weak features of the PS should be considered. On the other hand, PS-FH regions are mostly non-rigid and the coordinates of three points for AoP calculation are easily affected by the shape of segmented PS-FH. Therefore, the traditional rectangular convolution may be limited to model unknown deformations, especially boundaries. In this case, the effective receptive field of these networks will be reduced. In many studies (not only for PS-FH), super-pixel fusion (Ibrahim and El-kenawy, 2020) and region growing (Withey and Koles, 2007) have been used to provide prior information in deep networks, while spatial transform networks (Jaderberg et al., 2015) and deformable part models (Dai et al., 2017) are two advanced methods to solve the limitation of rectangle convolution kernels.

Therefore, a double branch segmentation network (DBSN) to consider weak features in PS-FH was proposed based on the UNet. The DBSN was composed of a shared encoder, a dual-branch decoder, a collaborative loss and parameter selection. In the dual-branch decoder, the upper branch was designed to extract all features for PS-FH segmentation, whereas the lower branch was designed to learn high-level semantic information (e.g., the shape of targets) further and distinguish targets (refinement). Between the lower decoding branch and the upper decoding branch, attention gates (AGs) were used to constrain the feature map input from the lower decoding branch to the upper decoding branch to learn more valuable features, while deformable convolution blocks in the decoding upper branch were also used to adapt to the geometric deformation of targets. The collaborative loss was proposed to effectively combine outputs of the upper and lower branches to enhance the weak features of PS-FH. In the optimization stage, critical parameters for the collaborative loss were selected. The significant contributions of this paper are summarized as follows:

- We proposed a new framework for AoP measurement. The framework includes image segmentation, target fitting and AoP calculation.
- We proposed a novel double branch network (DBSN) for PS-FH segmentation. In the dual-branch decoder, the lower branch with attention gates (AGs) provides high-level semantic information to refine the segmented areas of the upper branch.
- We introduced deformable convolution (DC) blocks to adapt to the geometric deformation of targets.
- We validated the effectiveness of the proposed methods using a small private dataset with 313 images and tested its generalization performance using a large public dataset with more than 3,700 images.

## 2 Materials and methods

In order to automatically compute AoP (Figure 1A), original TPU images were firstly preprocessed (Figure 1B), target areas (i.e., FH and PS) were secondly segmented with the proposed DBSN (Figure 1C), and these areas were thirdly fitted with elliptic equations and thereby three key points for computing AoP were determined (Figure 1D). Compared with GTs (Figure 1E), the performance of our method was evaluated with several metrics (Figure 1F).

### 2.1 Dataset

Experiments were conducted on our private dataset (Zhou et al., 2020) and the public JNU-IFM dataset (Lu et al., 2022b). Our private dataset was used to train and validate the proposed method, while the public JNU-IFM dataset was used to test its generalization performance.

In our private dataset, 313 TPU images with a resolution of 1,295 × 1,026 were collected from 84 patients by Zhou et al. (2020) to form a dataset for AoP calculation and were annotated by seven doctors with more than 10 years of ultrasound experience. Therefore, the dataset includes original TPU images and corresponding GTs that are composed of areas of FH and PS, the coordinates of the upper (*U*_*t*_) and lower (*L*_*t*_) endpoints of PS, PS’s long axis (*X*_*t*_) and manually measured AoP (*AoP*_*t*_) (Figure 1D). The 5-fold cross-validation procedure was conducted to split the training dataset into 5 folds. The first 4 folds were used to train our model and the holdout fifth fold was used as the test set. Since each patient had multiple TPU images, the data was randomly split so that all TPU images from each unique patient were only in one of the training and validation sets. This process was repeated and each of the folds was given an opportunity to be used as the holdout test set. The performance of our model was calculated as the mean of these runs.

In the public JNU-IFM dataset, 6,224 high-quality images with four categories were annotated using the Pair software and validated by two experienced radiologists. Over 3,700 images can be used to calculate AoP (Lu et al., 2022b). However, GTs include areas of FH and PS, but not manually measured AoP (*AoP*_*t*_). Therefore, pseudo labels of AoP were computed according to its definition based on the areas of FH and PS (Supplementary Data Sheet S1).

### 2.2 Pre-processing

For each original TPU image, its size was adjusted from 1,295 × 1,026 to 512 × 384 as well as its pixel values were normalized to [−1,1]. In order to increase the amount of data without affecting target segmentation and AoP calculation, these preprocessed images were randomly rotated by an angle between -30° and 30° and artificially flipped to generate new data. These new data were used to promote the training of the proposed model but did not lead to overfitting.

### 2.3 Image segmentation *via* the proposed double branch segmentation network

**FIGURE. 2:**
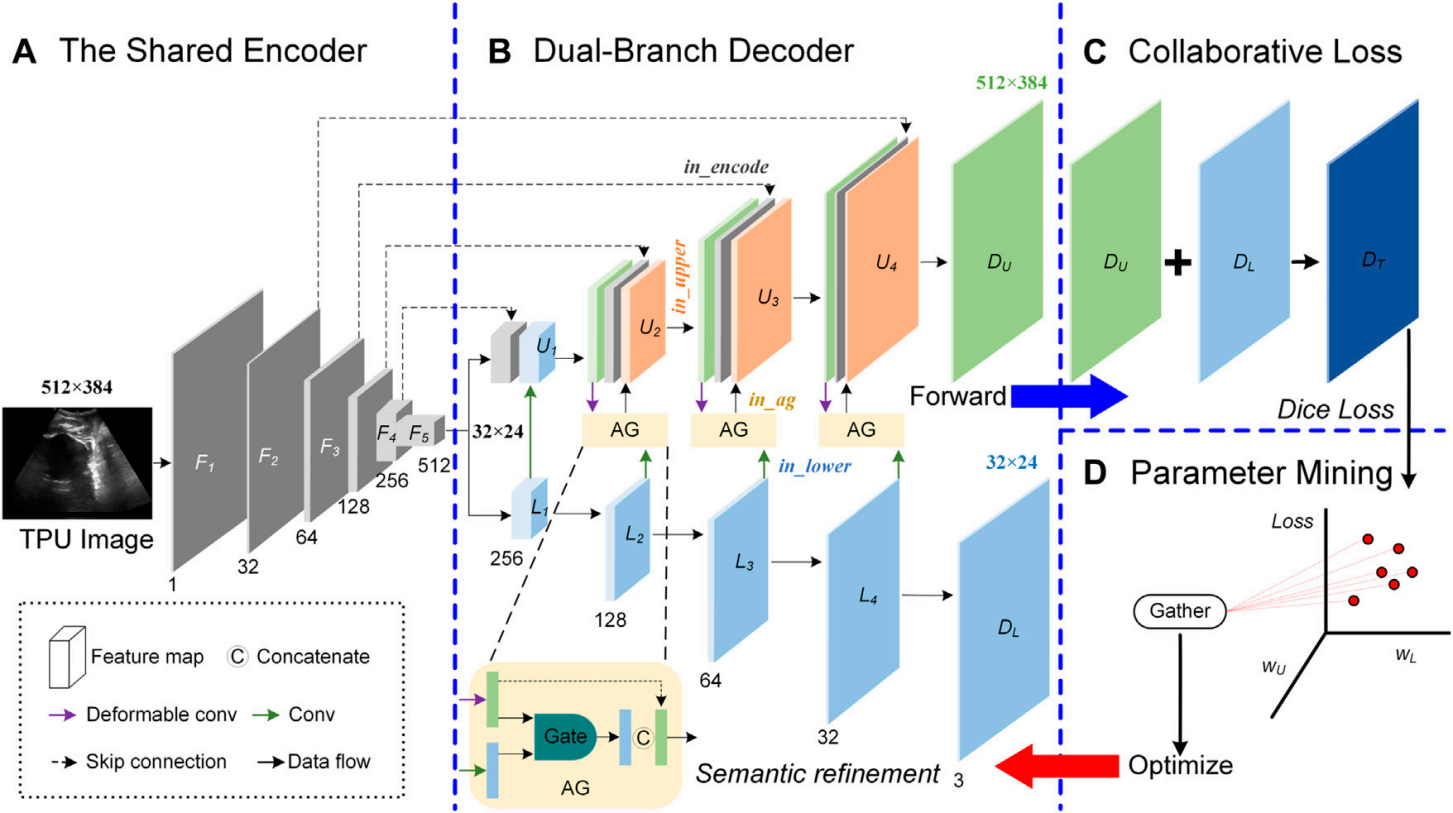
Overview of the proposed Double Branch Segmentation Network (DBSN) structure. This DBSN architecture is composed of a shared encoder **(A)**, a dual-branch decoder **(B)**, the collaborative loss **(C)** and the parameter mining **(D)**. In the dual-branch decoder, feature maps between the upper (U) and lower (L) branches are fused with attention gates (AGs). In each block of the upper (U) branch, feature maps come from the encoder (*In_encoder*), the upper decoder (*In_upper*) and the AG (*In_ag*) that consider the feature map of the lower decoder (*In_lower*).

In the process of acquiring the TPU image, the ultrasound probe cannot be accurately placed in a suitable place for a long time. Therefore, the target area in the acquired TPU image is blurred, the target boundary is not obvious and the target deformation is large. Other tissues or organs in the acquired TPU image further increase the difficulty of the segmentation task. In addition, the segmentation area in the TPU image has a large area and regular shape, and the small-area feature map containing high-level semantic information can also represent the segmentation area clearly. To accurately segment PS-FH, we proposed a double branch segmentation network (DBSN) to consider these weak features. Based on U-net, we added the decoding lower branch that performs decoding operations on the multi-channel small-area feature maps containing high-level semantic information. We used the decoded feature map of the decoding lower branch as the input of the decoding upper branch and made the decoding upper branch obtain higher-level semantic information. In addition, an attention mechanism was used to constrain the feature map input from the lower decoding branch to the upper decoding branch and thereby useful features in the feature map of the lower decoding branch can be learned by the upper decoding branch. Furthermore, in order to reduce the impact of the geometric deformation of the target region in the TPU image on the segmentation performance, we used deformable convolution blocks in the decoding upper branch to adapt to the geometric deformation of the input image. In detail, the proposed DBSN framework (Supplementary Table S1) is made up of four parts (Figure 2):

#### 2.3.1 Shared encoder

The shared encoder is made up of five convolution blocks denoted as [*F*_1_ *, F*_2_ *, F*_3_, *F*_4,_ and *F*_5_]. For *F*_1_, *F*_2_, *F*_3_, and *F*_4_, each block is followed by the 2 × 2 Max pooling and has two layers [each layer is composed of a 3 × 3 convolution operator (3 × 3 Conv), a group normalization (GN) and the rectified linear unit (ReLU)]. Different from *F*_1_, *F*_2_, *F*_3_, and *F*_4_, *F*_5_ is not followed by the 2 × 2 Max pooling. The output of the block *F*_*i*_ is the input of the block *F*_*i+*1_, but the number of channels of *F*_*i+*1_ is twice that of *F*_*i*_. Therefore, the size of feature map of *F*_5_ is 32 × 24 and its channel number is 512, as shown in Figure 2A.

#### 2.3.2 Dual-branch decoder

The input of the decoder is feature maps with a size of 32 × 24, its structure includes the upper branch (U) and the lower branch (L), and attention gates (AGs) are used to fuse feature maps between U and L, as shown in Figure 2B.

The lower branch (L) is composed of five convolution blocks denoted as [*L*_1_, *L*_2_, *L*_3_, *L*_4_, and *D*_*L*_]. For *L*_1_, *L*_2_, *L*_3,_ and *L*_4_, each block has two layers (each layer is composed of 3 × 3 Conv, GN and ReLU) and the channel number of its output is half of its input. *D*_*L*_ is made up of 1 × 1 Conv and the Softmax unit. The 1 × 1 Conv is used to reduce the channel number from 32 to 3, while the Softmax unit is used to generate a probability map of the three channels for areas of PS, FH and background. The size of feature map of *U*_*D*_ is 32 × 24.

The upper branch (U) consists of five blocks denoted as [*U*_1_, *U*_2_, *U*_3_, *U*_4_ and *D*_*U*_]. *U*_1_ has two parts: one comes from the output (denoted as *in_encode*) of *F*_4_ and the other one comes from the output (denoted as *in_lower*) of *L*_1_ after a series of processing (i.e., Up-sampling + 3 × 3 Conv + GN + ReLU). Each of the following three blocks (i.e., *U*_2_, *U*_3_ and *U*_4_) is made up of *in_encode*, the output (*in_upper*) of the former block after a series of processing (i.e., (3 × 3 Conv + GN + ReLU) × 2) and the output (*in_ag*) of the corresponding AG. The input of AG consists of *in_upper* after these processing (i.e., Up-sampling +3 × 3 Deformable Convolution (DC) + GN + ReLU) and *in_lower* after these processing (i.e., Up-sampling +3 × 3 Conv + GN + ReLU) at the same layer. Similar to *D*_*L*_, *D*_*U*_ is composed of 1 × 1 Conv and the Softmax unit, but the size of feature map of *D*_*U*_ is 512 × 384, as is shown in Figure 2B.

#### 2.3.3 Collaborative loss

The collaborative loss (*Loss*) fuses the two output branches of the proposed framework. The Dice (*D*) loss is used for each branch, as follows:

$$D=1-\frac{2\overset{N}{\underset{i=1}{\sum }}\overset{C}{\underset{j=1}{\sum }}y_{i,j}p_{i,j}}{\overset{N}{\underset{i=1}{\sum }}\overset{C}{\underset{j=1}{\sum }}\left(y_{i,j}+p_{i,j}\right)}$$ (1)

where *y* is the ground truth map, *p* is its corresponding predicted map, *N* is the number of pixels and *C* is the number of classes (excluding the background).

There are two decoder branches in the DBSN, so the total loss consists of two components-one for the lower branch (*D*_*L*_) and the other for the upper branch (*D*_*U*_), as shown in (Figure 2C).

$$Loss=w_{U}D_{U}+w_{L}D_{L}$$ (2)

where *w*_*U*_ and *w*_*L*_ are the weights for the upper and lower branches.

#### 2.3.4 Parameter mining

Parameters (*w*_*U*_ and *w*_*L*_) of the Eq. 2 were determined through a series of hyperparameter analysis. Although there are two decoder branches in the DBSN, the final segmented results are from the upper branch. Therefore, the loss for the upper branch (*D*_*U*_) is mainly used to optimize the network, whereas the loss for the lower branch only acts as an auxiliary optimization. In extreme cases, the network can be trained only with the loss from the upper branch (*D*_*U*_) and thereby $w_{U}$ is set to be one at the beginning. In order to assess the relative role of the upper (*D*_*U*_) and lower (*D*_*L*_) branches, we keep increasing the supporting effects of the lower (*D*_*L*_) branch without changing the upper (*D*_*U*_) branch by changing $w_{L}$ from 0.1 to 0.2, 0.3, 0.5 and 1.0. Effects of $w_{L}$ on the segmentation performance were evaluated with accuracy and Dice scores. As is shown in Supplementary Table S2, the best performance of DBSN is obtained when *w*_*L*_ is 0.2. Therefore, *w*_*U*_ = 1.0 and *w*_*L*_ = 0.2 are used in the proposed model, as shown in (Figure 2D).

### 2.4 Post-processing

In the present study, we only used the output of the upper branch. The output includes segmented regions of PS and FH. The ellipse contours of segmented regions were firstly fitted with the least square method (Gander et al., 1994). The ellipse equation is

$$F\left(a,x\right)=Ax^{2}+Bxy+Cy^{2}+Dx+Ey+F=0$$ (3)

where ***a*** = [*A B C D E F*]^*T*^ and ***x*** = [*x* ^2^ *xy y* ^2^ *x y* 1]^*T*^. *A B C D E F* are parameters, *x* and *y* are the coordinate horizontal and vertical positions.

The quadratic constraint (i.e., ***a*** ^*T*^ ***Ca*** = 1) is used to fit ellipses.

$$a^{T}\left[\begin{array}{l} \begin{array}{c} 0\\ 0\\ 2 \end{array}\\ \begin{array}{c} 0\\ 0\\ 0 \end{array} \end{array}\right.\left.\begin{array}{l} \begin{array}{c} 0\\ -1\\ 0 \end{array}\\ \begin{array}{c} 0\\ 0\\ 0 \end{array} \end{array}\begin{array}{l} \begin{array}{c} 2\\ 0\\ 0 \end{array}\\ \begin{array}{c} 0\\ 0\\ 0 \end{array} \end{array}\begin{array}{l} \begin{array}{c} 0\\ 0\\ 0 \end{array}\\ \begin{array}{c} 0\\ 0\\ 0 \end{array} \end{array}\begin{array}{l} \begin{array}{c} 0\\ 0\\ 0 \end{array}\\ \begin{array}{c} 0\\ 0\\ 0 \end{array} \end{array}\begin{array}{l} \begin{array}{c} 0\\ 0\\ 0 \end{array}\\ \begin{array}{c} 0\\ 0\\ 0 \end{array} \end{array}\right]a=1$$ (4)

The coordinates of the two endpoints (i.e., *U*_*p*_ and *L*_*p*_) of PS were secondly determined by the major axis (denoted as *X*_*p*_) of the elliptic curve of the area of PS. Thirdly, the right tangent (denoted as *T*_*p*_) connected to *L*_*p*_ was determined based on the elliptic curve of the area of FH. Finally, *AoP*_*p*_ is calculated with the three points (i.e., *U*_*p*_, *L*_*p*_, and *T*_*p*_), as is shown in Figure 1D.

### 2.5 Evaluation

In order to evaluate the proposed DBSN, different metrics were used for image segmentation, endpoint location and AoP calculation, as is shown in Figure 1F.

#### 2.5.1 Image segmentation

Accuracy (Acc), Dice scores and average surface distance (ASD) were used to evaluate the segmentation performance.

$$Acc=\frac{TP+TN}{TP+FP+TN+FN}$$ (5)

$$Dice=\frac{2TP}{2TP+FP+FN}$$ (6)

where *TP*, *FP*, *FN*, and *TN* denote true positive, false positive, false negative and true negative, respectively. In the present study, Dice scores include *Dice*_*PS*_ for segmented PS, *Dice*_*FH*_ for segmented FH and *Dice*_*all*_ for both targets.

Let *S*(*A*) denote the set of surface voxels of *A.* The shortest distance of an arbitrary voxel *v* to *S*(*A*) is defined as:

$$d\left(v,\;S\left(A\right)\right)=\begin{array}{c} \min\\ s_{A}\epsilon S\left(A\right) \end{array}\left\|v-s_{A}\right\|$$ (7)

where $\left\|.\right\|$ denotes the Euclidean distance. The ASD is then given by:

$$ASD\left(A,B\right)=\frac{1}{\left|S\left(A\right)\right|+\left|S\left(B\right)\right|}\left(\underset{s_{A}\epsilon S\left(A\right)}{\sum }d\left(s_{A},S\left(B\right)\right)+\underset{s_{B}\epsilon S\left(B\right)}{\sum }d\left(s_{B},S\left(A\right)\right)\right)$$ (8)

#### 2.5.2 Endpoint location of pubic symphysis

The Euclidean distance (ED) between the predicted endpoint coordinate and the corresponding GT’s coordinate and the angle (*A*_*x*_) between the predicted long axis (*X*_*p*_) and the GT’s long axis (*X*_*t*_) were used to evaluate the performance of different methods for endpoint location. Here, we assume that the predicted coordinates are *U*_*p*_ (*x*_*Up*_ *, y*_*Up*_) for the upper endpoint and *L*_*p*_ (*x*_*Lp*_ *, y*_*Lp*_) for the lower endpoint, and the predicted long axis is $\vec{X_{p}}=\left[x_{Lp}-x_{Up},y_{Lp}-y_{Up}\right]$ . The corresponding GTs are *U*_*t*_ (*x*_*Ut*_ *, y*_*Ut*_), *L*_*t*_ (*x*_*Lt*_ *, y*_*Lt*_) and $\vec{X_{t}}=\left[x_{Lt}-x_{Ut},y_{Lt}-y_{Ut}\right]$ . Therefore, *ED*_*U*_, *ED*_*L*_ and *Ax* are computed as follows:

$$ED_{U}={[{(x_{Ut}-x_{Up})}^{2}+{(y_{Ut}-y_{Up})}^{2}]}^{0.5}$$ (9)

$$ED_{L}={[{(x_{Lt}-x_{Lp})}^{2}+{(y_{Lt}-y_{Lp})}^{2}]}^{0.5}$$ (10)

$$A_{x}=\cos^{-1}\frac{\vec{X_{p}}∙\vec{X_{t}}}{\left|\vec{X_{p}}\right|\left|\vec{X_{t}}\right|}$$ (11)

#### 2.5.3 Angle of progression calculation

The AoP difference ( ∆ AoP) between the predicted AoP (*AoP*_*p*_) and the GT’s AoP (*AoP*_*t*_) was used to evaluate the performance of different approaches for AoP calculation.

$$∆AoP=\left|{AoP}_{p}-{AoP}_{t}\right|$$ (12)

here, mean ( ∆ AoP_Mean), median ( ∆ AoP_Median) and standard deviation ( ∆ AoP_Std) of ∆AoP also are used as evaluation metrics.

### 2.6 Experimental setup

Based on PyTorch, the methods investigated in the present study are run on an E5-2680 v4 CPU system with 128 GB memory and an NVIDIA GTX2080Ti GPU. The learning rate is set to be 0.0001 and the Kaiming algorithm is used to initialize the network weights. The proposed model is trained for 200 epochs with a batch size of 1 and evaluated with 5-fold cross-validation (Fushiki, 2011). The final score is generally the average of all the scores obtained across the 5-folds.

## 3 Results

Three sets of comparative experiments were designed to illustrate the effectiveness of our method. The role of different parts of the DBSN in feature extraction was investigated (Section 3.1), the performance improvement of key components used in our model was quantified in the first sets of comparative experiments (Section 3.2), the generalization performance of the proposed models was also evaluated on the public dataset (Section 3.3) and comparison of our method with the existing deep learning approach was also performed in Section 3.4.

### 3.1 Feature maps in different branches of the double branch segmentation network

**FIGURE 3:**
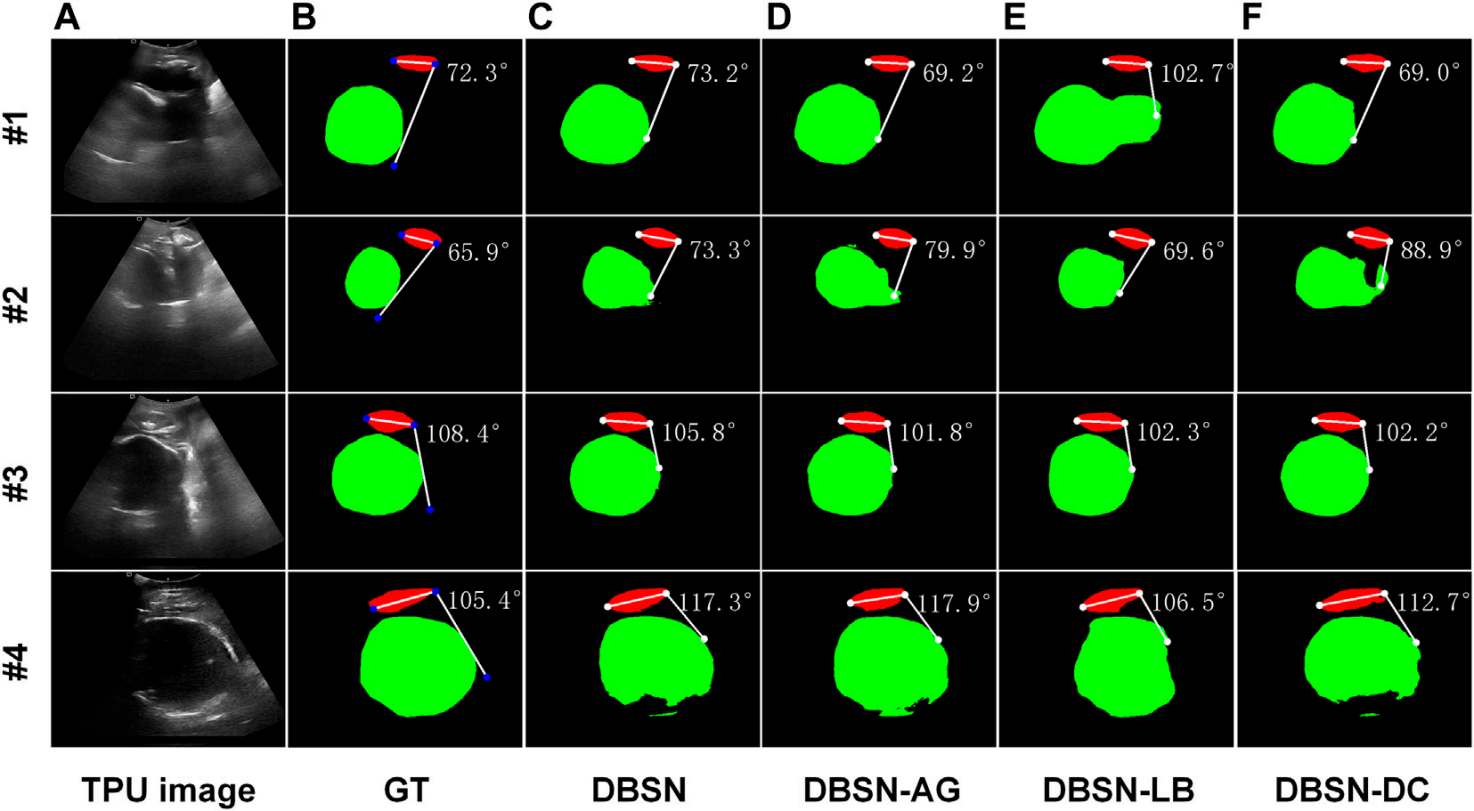
Four examples (#1, #2, #3 and #4) of segmentation and AoP calculation. From left to right, original images as input **(A)**, ground truth (GT, **(B)** and results of four different networks are shown. DBSN **(C)**, DBSN-AG **(D)**, DBSN -LB **(E)** and DBSN-DC **(F)** denotes, double branch segmentation network (DBSN), DBSN without the attention gate (AG), DBSN without the lower branch (LB) and DBSN without deformable convolution blocks (DC), respectively.

As is shown in the proposed DBSN (Figure 2), there are four important parts: a shared encoder (*In_encoder*), a dual-branch decoder (including the upper (*In_upper*) and lower (*In_lower*) branches) and AGs (*In_ag*) used for fusing feature maps between two decoder branches. To investigate the role of four parts (i.e., *In_encoder, In_upper, In_lower* and *In_ag*) in feature extraction, the learned feature maps in the level with 32 channels are shown in Supplementary Figure S1. Given a randomly selected TPU image (Supplementary Figure S1A), four examples of feature maps of each part (i.e., *In_encoder, In_upper, In_lower* or *In_ag*) (Supplementary Figure S1B) are compared to the corresponding GT (Supplementary Figure S1C). The feature maps of *In_encoder* (Figure 3Bi) contain most of the details of the original TPU image (Supplementary Figure S1A). Different from feature maps of *In_encoder* (Supplementary Figure S1Bi)*,* each of the feature maps of *In_upper* only includes part information of the original TPU image, and features for FH, PS and the background are separated from the original TPU image (Supplementary Figure S1Bii). Different from features of *In_upper* (Supplementary Figure S1Bii), features maps of *In_lower* only contain FH and (or) PS, the deformation of target areas (i.e., FH and PS) is larger, and their detailed information is not retained (the contours of the target regions are relatively irregular and it is almost impossible to observe the Fan-shaped contour of the original TPU image) (Supplementary Figure S1Biii). Compared with feature maps of *In_upper* (Supplementary Figure S1Bii) and *In_lower* (Supplementary Figure S1Biii), areas of FH and (or) PS of feature maps of *In_ag* (Supplementary Figure S1Biv) are more obvious. Detailed information of FH and PS of *In_ag* is more than that of *In_lower* but less than that of *In_upper*. Moreover, the contours of the target areas similar to ellipses are close to that of GT (Supplementary Figure S1C). Therefore, micro (*In_upper*) and macro (*In_lower*) semantic information can be fused to generate feature maps (*In_ag*) similar to GT. These results are associated with key components of the DBSN and should be further investigated.

### 3.2 The performance improvement of key components

In order to explain the effectiveness of the proposed method, we designed an ablation experiment to investigate the effects of key components of DBSN on the automatic measurement of AoP. As is shown in Supplementary Figure S2, four different architectures are the proposed DBSN model with the lower branch (LB), attention gate (AG) and deformable convolution (DC) (Supplementary Figure S2A), DBSN without AG (DBSN-AG) (Supplementary Figure S2B), DBSN without LB (DBSN-LB) (Supplementary Figure S2C) and DBSN without DC (DBSN-DC) (Supplementary Figure S2D). The performance of the four models is listed as follows:

Segmentation performance of these models on Acc, Dice_all_, Dice_PS_ and Dice_FH_ presents in Supplementary Table S3. Compared with the results of DBSN-AG, Dice_PS_ of DBSN is slightly improved, indicating that the AG module increases the accuracy of segmented PS of the upper branch of the DBSN. Compared with the results of DBSN-LB, Acc, Dice_all_ and Dice_FH_ of M are increased, whereas Dice_PS_ of DBSN is reduced. These results suggested that *LB* can provide contour information for the upper branch and thereby the overall segmentation accuracy (i.e., Acc, Dice_all_ and Dice_FH_) is increased. In addition, feature maps of *LB* can interfere with the segmentation of PS by the upper branch (Supplementary Figure S1B), resulting in a decrease in Dice_PS_ *.* Compared with the results of DBSN-DC, the performance of DBSN on all metrics are improved and these results demonstrated that DC could comprehensively improve segmentation performance compared with traditional convolution blocks. In summary, DC is suitable to deal with the data samples used in the present study and AG can improve the anti-interference ability of the upper branch (Supplementary Figure S1B) and enhance the robustness of our model. The *LB* has a double effect. On the one hand, it provides high-level semantic information and promotes the segmentation performance of the upper branch. On the other hand, it brings interference that is not related to the decoding task of the upper branch.

The performance of DBSN, DBSN-AG, DBSN-LB and DBSN-DC on the computed accuracy of the upper and lower endpoints of PS was further investigated. The computed accuracy of endpoints of PS is associated with PS’s segmented accuracy evaluated with Dice_PS_. The higher Dice_PS_ (Supplementary Table S3), the smaller *ED*_*U*_, *ED*_*L*_, and *A*_*X*_ (Supplementary Table S4). In the four models, the largest Dice_PS_ (Supplementary Table S3) and the smallest *A*_*X*_ (Supplementary Table S4) are obtained for the DBSN-LB model. Especially, Dice_PS_ of DBSN-DC is lower than that of DBSN-AG (Supplementary Table S3), but DBSN-DC’s *A*_*X*_ is larger than that of DBSN-AG (Supplementary Table S4). The results demonstrated that the segmented PS by the upper branch is more susceptible to interference from LB, resulting in the deformation of the segmented PS (Supplementary Figure S1B) and thereby larger *A*_*X*_.

We also investigated the performance of DBSN, DBSN-AG, DBSN-LB and DBSN-DC on the computed accuracy of AoP. The computed accuracy of AoP depends on three key points (i.e., two endpoints of PS and the tangent point of FH contour) (Figure 1D). As is shown in Figure 3, the accuracy of FH segmentation partly determines AoP calculation. In detail, DBSN performs the best in cases #1 and #3, whereas DBSN-LB performs the best in cases #2 and #4. Moreover, DBSN-AG for cases #3 and #4, DBSN-LB for the case #1, and DBSN-DC for the case #2 perform the worst, respectively. Similarly, the accuracy and robustness of the DBSN model in AoP measurement are better than other models, especially for ∆ AoP_Mean and ∆ AoP_Std (Supplementary Table S5). DBSN-LB has the best performance in ∆ AoP_Median and the second rank in ∆ AoP_Mean. Compared with DBSN-AG (DBSN with LB but without AG), the better ∆ AoP_Median and ∆ AoP_Mean of DBSN-LB (DBSN without LB and AG) is obtained. The results indicated that adding LB cannot directly improve the performance of the AoP measurement. Compared with DBSN-LB (DBSN without LB and AG), DBSN contains LB and AG, and has better performance, showing the combination of LB and AG can improve the accuracy of the AOP measurement.

In summary, the performance of the DBSN model on all metrics is not the best (Supplementary Tables S3–S5), but its advantage is that it maintains not only high segmentation accuracy (Supplementary Table S3) but also has obvious advantages in AoP calculation (Supplementary Table S5). Its robustness is reflected by avoiding the extreme cases of target segmentation, and stable AoP calculation with lower ∆ AoP_Mean and ∆ AoP_Std.

### 3.3 Comparison of different deep learning methods

To further investigate the effectiveness of DBSN used in the present study, the results of DBSN are compared with other methods used for AoP calculation. These methods include U-net, attention U-net (attU-net), OTSU and the multitask attention fusion network (MTAFN) (Zhou et al., 2020). Results of image segmentation of different methods are listed in Table 1. The proposed DBSN performs the best by achieving 98.7% of Acc, 93.4% of Dice_all_, 91.0% of Dice_PS_ and 93.7% of Dice_FH_. The best segmentation performance makes DBSN accurately compute AoP, resulting in the smallest difference (i.e., ∆ AoP_Mean) between predicted AoP and GT. This high segmentation accuracy comes at the cost of higher model complexity and more inference time. The most complex model is MTAFN, where the model with the longest inference time is DBSN. As is listed in Table 2, the proposed DBSN outperforms other methods by achieving the smallest ∆ AoP_Mean (5.993°) and ∆ AoP_Std (3.872°), but its ∆ AoP_Median is slightly larger than that of MTAFN and ranks second. Therefore, the above results indicate that the proposed DBSN can effectively improve the AoP measurement by segmenting FH and PS.

**TABLE 1:** Comparison of segmentation results of different algorithms.

Model	Acc	Dice_all_	Dice_PS_	Dice_FH_	Size (M)	Time (ms)
MTAFN	0.982	0.907	0.901	0.907	13.08	16.56
STU	0.980	0.895	0.887	0.898	—	—
U-net	0.984	0.913	0.889	0.916	8.63	9.36
AttU-net	0.984	0.914	0.900	0.916	8.72	11.43
DBSN	0.987	0.934	0.910	0.937	10.34	76.80

**TABLE 2:** Comparison of computed AoPs of different algorithms.

Model	∆ AoP_Mean (°)	∆ AoP_Median (°)	∆ AoP_Std (°)
MTAFN	7.60	**4.68**	8.85
STU	9.26	6.03	10.22
U-net	8.00	5.97	7.11
AttU-net	7.50	5.99	6.33
DBSN	**5.993**	5.851	**3.872**

The best results are marked with the bold values.

### 3.4 The generalization performance of the proposed method

To verify the generalization of the proposed method, its performance was evaluated by using the independent test dataset (JUN-IFM). Here, we took the public dataset with >3,700 images as an independent test dataset, that is, these models are only trained on our private dataset with 313 images and then tested on the independent test dataset. For these models’ training, we used 5-fold cross-validation to train these models with our private dataset, so we got five models. The final result on JUN-IFM is the average of all the scores of these five models. The segmentation performance of four models (DBSN, DBSN-AG, DBSN-LB and DBSN-DC) on Dice_all_, Dice_PS_ and Dice_FH_ presents in Supplementary Table S6. The best results show the values of Dice_all_, Dice_PS_ and Dice_FH_ are 91.9% for DBSN-AG, 87.2% for DBSN-LB and 92.4% for DBSN-DC, respectively. The performance of these four models on the computed accuracy of AoP was further evaluated. As is listed in Supplementary Table S7, the computed AoP, ∆ AoP_Mean, ∆ AoP_Median and ∆ AoP_Std, respectively, reach 5.110° for DBSN-DC, 4.181° for DBSN-DC, 4.338° for DBSN-LB. Compared to the performance of the proposed method (i.e., DBSN model) on our private dataset (Supplementary Tables S3–S5), its performance on the public JUN-IFM dataset shows that both segmentation and AoP calculation accuracy are slightly degraded (Table 3). In detail, Diceall, DicePS and DiceFH reduced by 1.7% (from 93.38% to 91.8%), 4.1% (from 91.01% to 87.26%) and 1.4% (from 93.66% to 92.34%), respectively. ASD increased from 6.268 to 7.729 pixels. ∆ AoP_Mean decreased 0.82° (from 5.993° to 5.178°).

**TABLE 3:** Performance of different methods on the public JNU-IFM dataset.

Model	Dice_all_	Dice_PS_	Dice_FH_	ASD	∆ AoP_Mean (°)
U-net	0.8667	0.8160	0.8730	12.693	8.900
AttU-net	0.8696	0.8219	0.8728	11.754	7.896
DBSN	0.9180	0.8726	0.9234	7.729	5.178

## 4 Discussion

Monitoring FH descent is important for taking necessary interventions in time. Although the digital examination is a traditional method, its limited accuracy and possible harm to pregnant women limit its application (Rozenberg et al., 2004). Recently, AoP as the more accurate parameter has been suggested to provide the best diagnosis and management of a woman in labor. Segmentation of PS-FH is crucial to automatically measure AoP, but is challenging because of missing boundaries, low signal-to-noise ratio, the speckle pattern, etc.

This present study is one of the few that implements the automatic measurement of AoP based on data-driven deep learning methods. The proposed framework includes two stages: PS-FH segmentation at the first stage and determination of three key points for the AoP measurement at the second stage. At the first stage, the upper branch of the proposed DBSN outputs the segmentation results, and the lower branch with DC blocks and AGs provides the high-level semantic information to refine the segmented areas of the upper branch. At the second stage, segmented areas were ellipse-fitted and thereby coordinates of three key points (including the endpoints of the long axis of PS and the right tangent point of FH) were calculated for the AoP measurement. In the all-existing approaches, most studies have relied heavily on manual measurement, while a few studies attempted to automatically measure AoP (Conversano et al., 2017; Angeli et al., 2020a; Angeli et al., 2020b). Conversano et al. (2017) combined morphological filters with pattern recognition methods to identify PS and FH, and segmented targets were used to calculate AoP. Similarly, Montaguti et al. (2018) identified PS-FH and then measured AoP with a novel software (Sono Labor & Delivery, GE Medical Systems, Zipf, Austria). These approaches required a shape prior for initialization, but this initialization was either based on assumptions from observing the TPU images or manually generated. In contrast to the above two methods, our approach does not need additional information apart from TPU images or manual initialization for selecting ideal images. Furthermore, the results of our approach showed high accuracy of the PS-FH segmentation as well as significant improvements of the AoP calculation.

The high accuracy of the AoP calculation may be attributed to the PS-FH segmentation based on deep learning approaches. Due to the straightforward, efficient and accurate characteristics, deep learning approaches are widely used for TPU image segmentation and classification (Drukker et al., 2020; Xie et al., 2020). Using convolutional neural networks in many medical image segmentation tasks, excellent segmentation results have been achieved (LeCun et al., 2015). However, due to all semantic information learned by the above networks, they lack the ability to focus on the problem-oriented information-an aspect DBSN excels at. The AoP measurement is based on the shape of PS-FH and thereby the proposed method for PS-FH segmentation should consider its ellipse-like shape. In this aspect, the proposed DBSN is designed with a dual-pathway in the decoding part, higher-level semantic information about shape features is provided by the lower decoding branch for the upper decoding branch, and DC blocks and the AGs are used to capture ellipse-like shape features and help the decoding upper branch focus on more effective feature regions. These modifications to Unet result in superior performance (Supplementary Table S8), however, these modifications increase the model complexity by 19.8% and the inference time by 721%.

Although the proposed method was trained and validated on our small private dataset with 313 TPU images (Zhou et al., 2020) and good results were obtained, its generalization performance was also evaluated on the public JNU-IFM dataset with more than 3,700 TPU images that meet the requirements of AoP measurement (Lu et al., 2022b) and the Dice coefficient (Diceall) still exceeded 91% in the case of slight decrease (<2%). The reduction of Diceall is mainly due to the decrease of segmentation accuracy of PS. DicePS significantly reduced by 4.1%, whereas DiceFH decreased only slightly by 1.4%. Therefore, more attention needs to be paid to the PS segmentation in the future. Nevertheless, the proposed method trained on a small dataset can obtain such performance on the public large dataset, illustrating that the DBSN is a robust approach for PS-FH segmentation.

**FIGURE 4:**
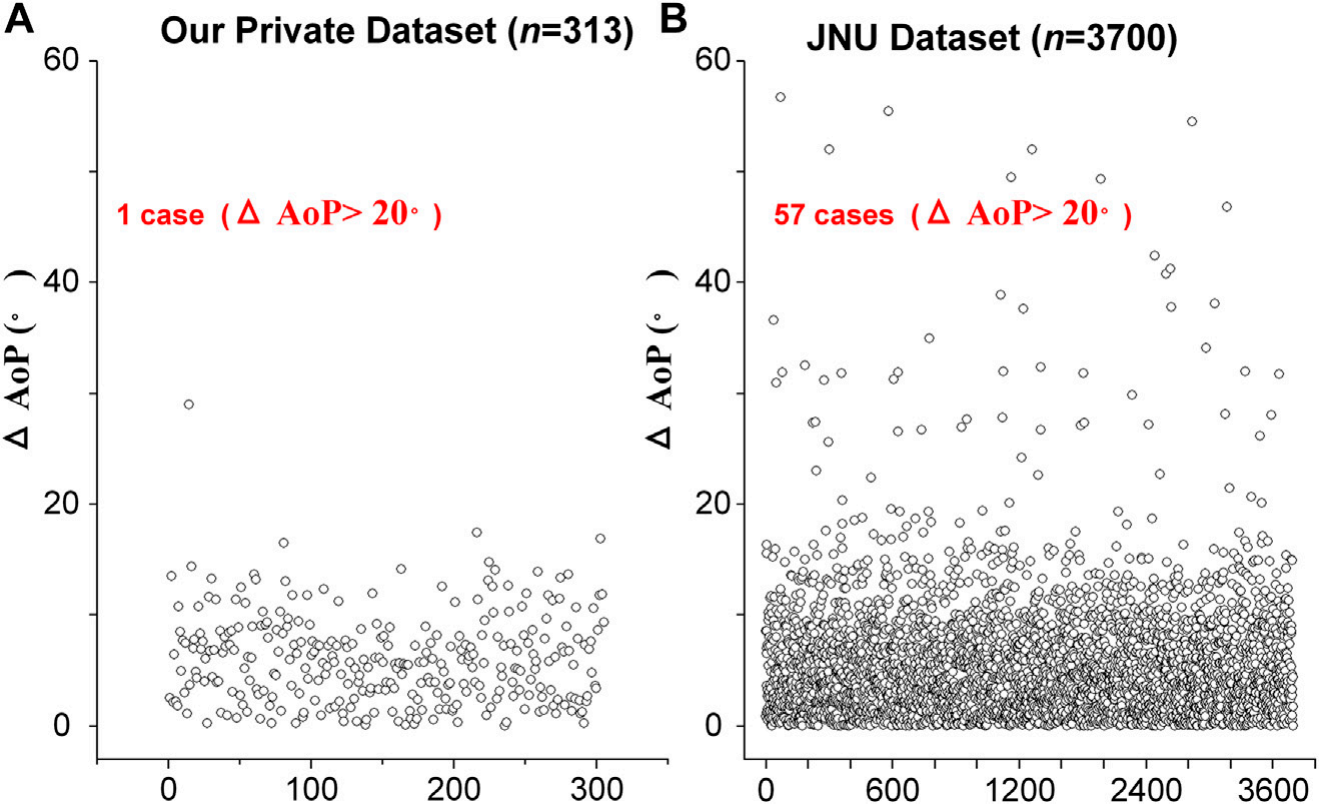
The distribution of AoP difference ( ∆ AoP) between the predicted AoP and the GT’s AoP. **(A)** There is one case with ∆ AoP > 20° in our private dataset (*n* = 313). **(B)** 57 cases with ∆ AoP >20° in the public dataset (*n* > 3,700).

**FIGURE 5:**
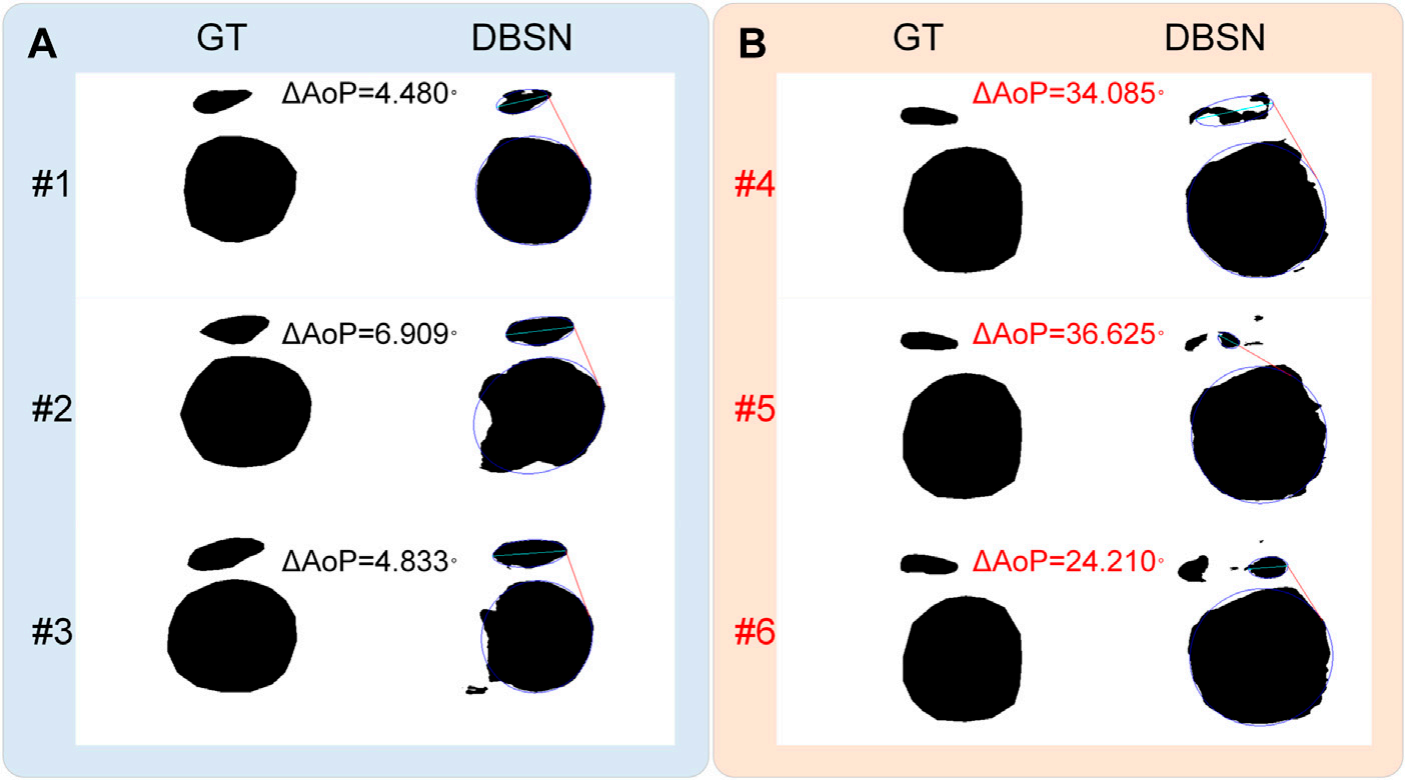
Effects of incorrect results on the AoP measurement. **(A)** Incorrect segmented results (#1, #2 and #3) have little effect on the determination of the three points, leading to a smaller ∆ AoP (<20°). **(B)** Incorrect segmented results (#4, #5 and #6) have a significant impact on the determination of any of the three points, resulting in a larger ∆ AoP (>20°).

According to the distribution of ∆ AoP, there is one case with ∆ AoP >20° in our private dataset (*n* = 313), whereas 57 cases with ∆ AoP >20° in the public dataset (*n* = 3,700) are found (Figure 4). The reason for this difference is partly that our model is trained and validated on our private dataset (*n* = 313) and tested on the public dataset (*n* = 3,700) without training. In addition, if incorrect segmented results have little effect on the determination of the three points (i.e., two endpoints of PS and the tangent point of FH contour) and a smaller ∆ AoP (<20°) is obtained. If incorrect segmented results (#4, #5 and #6) have a significant impact on the determination of any of the three points, resulting in a larger ∆ AoP (>20°) (Figure 5). As is shown in Figure 5A, the contour of the right part of the fetal head is the key to determine the tangent point for AoP measurement, and an incorrect segmented result of the left side of the fetal head has little effect on ∆ AoP. However, if incorrect segmented results are present in the right part of PS (related to the right endpoint), the left part of PS (related to the left endpoint) and (or) the right part of FH (related to the right tangent point), a larger ∆ AoP (>20°) is obtained. Therefore, further studies should pay attention to the relationship between target segmentation and key point identification.

Although DBSN has achieved good results, limitations and possibilities in the future include: 1) Our method is tested on a dataset from one center, so its effectiveness should be further verified on more multi-center datasets; 2) The JNU-IMF dataset only provided labels for target segmentation, and we used these labels to compute AoP as pseudo labels for AoP prediction (Supplementary Data Sheet S1). Special attention should be paid to the part of evaluating the performance of our method for AoP prediction on the JNU-IMF dataset. 3) Compared with traditional convolution blocks, DC blocks require more computing resources that will limit the application of our method in medical equipment (Dai et al., 2017); and 4) Inspired by the method of Conversano et al. (2017), the relevance between images in the same patient can be considered if our method will be applied to the real-time monitoring of AoP.

## 5 Conclusion

This work studies the automatic measurement method of AoP, and proposes a DBSN model for PS-FH segmentation from TPU images. In the DBSN, DC blocks are adapted to consider the geometric deformation of the data samples, the decoding branches are designed to make the lower decoding branch provide higher-level semantic information for the upper decoding branch, and the AG is used to constrain the feature map of the lower decoding branch to help the decoding upper branch focus on more effective feature regions. Comprehensive ablation experiments and comparative experiments demonstrated the proposed approach can effectively segment the target regions and is more suitable for the automatic measurement of AoP based on the ellipse fitting algorithm. In conclusion, our method is an important step toward the AoP measurement based on deep learning.

## Data Availability

The original contributions presented in the study are included in the article/Supplementary Material, further inquiries can be directed to the corresponding authors.

## References

[B1] Angeli L. Conversano F. Dall'asta A. Eggebø T. Volpe N. Marta S. (2020a). Automatic measurement of head-perineum distance during intrapartum ultrasound: Description of the technique and preliminary results. J. Matern. Fetal. Neonatal Med. 35, 2759–2764. 10.1080/14767058.2020.1799974 32727248

[B2] Angeli L. Conversano F. Dall'asta A. Volpe N. Simone M. Di Pasquo E. (2020b). New technique for automatic sonographic measurement of change in head–perineum distance and angle of progression during active phase of second stage of labor. Ultrasound Obstet. Gynecol. 56, 597–602. 10.1002/uog.21963 31909525

[B3] Barbera A. F. Pombar X. Perugino G. Lezotte D. C. Hobbins J. C. (2009). A new method to assess fetal head descent in labor with transperineal ultrasound. Ultrasound Obstet. Gynecol. 33, 313–319. 10.1002/uog.6329 19248000

[B4] Boyle A. Reddy U. M. Landy H. J. Huang C. C. Driggers R. W. Laughon S. K. (2013). Primary cesarean delivery in the United States. Obstet. Gynecol. 122, 33–40. 10.1097/AOG.0b013e3182952242 23743454 PMC3713634

[B5] Brunelli E. Youssef A. Soliman E. M. Del Prete B. Mahmoud M. H. Fikry M. (2021). The role of the angle of progression in the prediction of the outcome of occiput posterior position in the second stage of labor. Am. J. Obstet. Gynecol. 225, 81.e1. 10.1016/j.ajog.2021.01.017 33508312

[B6] Burgos-Artizzu X. P. Coronado-Gutiérrez D. Valenzuela-Alcaraz B. Bonet-Carne E. Eixarch E. Crispi F. (2020). Evaluation of deep convolutional neural networks for automatic classification of common maternal fetal ultrasound planes. Sci. Rep. 10, 10200. 10.1038/s41598-020-67076-5 32576905 PMC7311420

[B7] Cohen S. M. Lipschuetz M. Yagel S. (2017). Is a prolonged second stage of labor too long? Ultrasound Obstet. Gynecol. 50, 423–426. 10.1002/uog.17563 28640477

[B8] Conversano F. Peccarisi M. Pisani P. Di Paola M. De Marco T. Franchini R. (2017). Automatic ultrasound technique to measure angle of progression during labor. Ultrasound Obstet. Gynecol. 50, 766–775. 10.1002/uog.17441 28233418

[B9] Dai J. Qi H. Xiong Y. Li Y. Zhang G. Hu H. (2017). “Deformable convolutional networks,” in Proceedings of the IEEE international conference on computer vision), Venice, Italy, 22-29 October 2017, 764–773.()

[B10] Dall'asta A. Angeli L. Masturzo B. Volpe N. Schera G. B. L. Di Pasquo E. (2019). Prediction of spontaneous vaginal delivery in nulliparous women with a prolonged second stage of labor: The value of intrapartum ultrasound. Am. J. Obstet. Gynecol. 221, e641–e642. e613. 10.1016/j.ajog.2019.09.045 31589867

[B11] Drukker L. Noble J. A. Papageorghiou A. T. (2020). Introduction to artificial intelligence in ultrasound imaging in obstetrics and gynecology. Ultrasound Obstet. Gynecol. 56, 498–505. 10.1002/uog.22122 32530098 PMC7702141

[B12] Dückelmann A. M. Bamberg C. Michaelis S. A. Lange J. Nonnenmacher A. Dudenhausen J. W. (2010). Measurement of fetal head descent using the 'angle of progression' on transperineal ultrasound imaging is reliable regardless of fetal head station or ultrasound expertise. Ultrasound Obstet. Gynecol. 35, 216–222. 10.1002/uog.7521 20069668

[B13] Dupuis O. Ruimark S. Corinne D. Simone T. André D. René-Charles R. (2005). Fetal head position during the second stage of labor: Comparison of digital vaginal examination and transabdominal ultrasonographic examination. Eur. J. Obstet. Gynecol. Reprod. Biol. 123, 193–197. 10.1016/j.ejogrb.2005.04.009 15925438

[B14] Fitzpatrick M. Mcquillan K. O'herlihy C. (2001). Influence of persistent occiput posterior position on delivery outcome. Obstet. Gynecol. 98, 1027–1031. 10.1016/s0029-7844(01)01600-3 11755548

[B15] Fushiki T. (2011). Estimation of prediction error by using K-fold cross-validation. Stat. Comput. 21, 137–146. 10.1007/s11222-009-9153-8

[B16] Gander W. Golub G. H. Strebel R. (1994). Least-squares fitting of circles and ellipses. BIT Numer. Math. 34, 558–578. 10.1007/bf01934268

[B17] Ghi T. Farina A. Pedrazzi A. Rizzo N. Pelusi G. Pilu G. (2009). Diagnosis of station and rotation of the fetal head in the second stage of labor with intrapartum translabial ultrasound. Ultrasound Obstet. Gynecol. 33, 331–336. 10.1002/uog.6313 19202576

[B18] Hamilton E. F. Simoneau G. Ciampi A. Warrick P. Collins K. Smith S. (2016). Descent of the fetal head (station) during the first stage of labor. Am. J. Obstet. Gynecol. 214, 360.e361–e6. 10.1016/j.ajog.2015.10.005 26475422

[B19] Ibrahim A. El-Kenawy E.-S. M. (2020). Applications and datasets for superpixel techniques: A survey. J. Comput. Sci. Inf. Syst. 15.

[B20] Jaderberg M. Simonyan K. Zisserman A. (2015). Spatial transformer networks. Adv. neural Inf. Process. Syst. 28, 2017–2025.

[B21] Kalache K. D. Dückelmann A. M. Michaelis S. A. Lange J. Cichon G. Dudenhausen J. W. (2009). Transperineal ultrasound imaging in prolonged second stage of labor with occipitoanterior presenting fetuses: How well does the 'angle of progression' predict the mode of delivery? Ultrasound Obstet. Gynecol. 33, 326–330. 10.1002/uog.6294 19224527

[B22] Lecun Y. Bengio Y. Hinton G. (2015). Deep learning. Nature 521, 436–444. 10.1038/nature14539 26017442

[B23] Lu Y. Zhi D. Zhou M. Lai F. Chen G. Ou Z. (2022a). Multitask deep neural network for the fully automatic measurement of the angle of progression. Comput. Math. Methods Med. 2022, 5192338. 10.1155/2022/5192338 36092792 PMC9462992

[B24] Lu Y. Zhou M. Zhi D. Zhou M. Jiang X. Qiu R. (2022b). Corrigendum to "The JNU-IFM dataset for segmenting pubic symphysis-fetal head" data in brief. 41 (2022) 107904. Data Brief. 41, 108128. 10.1016/j.dib.2022.108128 PMC9006759 35434222

[B25] Montaguti E. Rizzo N. Pilu G. Youssef A. (2018). Automated 3D ultrasound measurement of the angle of progression in labor. J. Matern. Fetal. Neonatal Med. 31, 141–149. 10.1080/14767058.2016.1277701 28043183

[B26] Oboro V. O. Tabowei T. O. Bosah J. O. (2005). Fetal station at the time of labour arrest and risk of caesarean delivery. J. Obstet. Gynaecol. 25, 20–22. 10.1080/01443610400022512 16147687

[B27] Rozenberg P. Rudant J. Chevret S. Boulogne A. I. Ville Y. (2004). Repeat measurement of cervical length after successful tocolysis. Obstet. Gynecol. 104, 995–999. 10.1097/01.AOG.0000143254.27255.e9 15516390

[B28] Segel S. Y. Carreño C. A. Weiner S. J. Bloom S. L. Spong C. Y. Varner M. W. (2012). Relationship between fetal station and successful vaginal delivery in nulliparous women. Am. J. Perinatol. 29, 723–730. 10.1055/s-0032-1314895 22644826 PMC4091771

[B29] Sherer D. M. Miodovnik M. Bradley K. S. Langer O. (2002). Intrapartum fetal head position I: Comparison between transvaginal digital examination and transabdominal ultrasound assessment during the active stage of labor. Ultrasound Obstet. Gynecol. 19, 258–263. 10.1046/j.1469-0705.2002.00641.x 11896947

[B30] Simkin P. (2010). The fetal occiput posterior position: State of the science and a new perspective. Birth 37, 61–71. 10.1111/j.1523-536X.2009.00380.x 20402724

[B31] Withey D. J. Koles Z. J. (2007). “Medical image segmentation: Methods and software,” in 2007 Joint Meeting of the 6th International Symposium on Noninvasive Functional Source Imaging of the Brain and Heart and the International Conference on Functional Biomedical Imaging, Hangzhou, China, 12-14 October 2007 (IEEE), 140–143.()

[B32] Xie H. N. Wang N. He M. Zhang L. H. Cai H. M. Xian J. B. (2020). Using deep-learning algorithms to classify fetal brain ultrasound images as normal or abnormal. Ultrasound Obstet. Gynecol. 56, 579–587. 10.1002/uog.21967 31909548

[B33] Youssef A. Brunelli E. Azzarone C. Di Donna G. Casadio P. Pilu G. (2021). Fetal head progression and regression on maternal pushing at term and labor outcome. Ultrasound Obstet. Gynecol. 58, 105–110. 10.1002/uog.22159 32730691

[B34] Youssef A. Maroni E. Ragusa A. De Musso F. Salsi G. Iammarino M. T. (2013). Fetal head-symphysis distance: A simple and reliable ultrasound index of fetal head station in labor. Ultrasound Obstet. Gynecol. 41, 419–424. 10.1002/uog.12335 23124698

[B35] Zhang J. Landy H. J. Ware Branch D. Burkman R. Haberman S. Gregory K. D. (2010). Contemporary patterns of spontaneous labor with normal neonatal outcomes. Obstet. Gynecol. 116, 1281–1287. 10.1097/AOG.0b013e3181fdef6e 21099592 PMC3660040

[B36] Zhou M. Yuan C. Chen Z. Wang C. Lu Y. (2020). “Automatic angle of progress measurement of intrapartum transperineal ultrasound image with deep learning,” in International conference on medical image computing and computer-assisted intervention (Berlin, Germany: Springer), 406–414.

