# Supplementary material for: A framework for computing angle of progression from transperineal ultrasound images for evaluating fetal head descent using a novel double branch network
Source: Front Physiol. 2022 Dec 2;13:940150. doi: 10.3389/fphys.2022.940150 (PMC9755498; doi:10.3389/fphys.2022.940150)
Supplement: Supplementary file 1 [file Table1.docx]

**Supplementary information**

Jieyun Bai^1,2,^*, Zhanhang Sun^1^, Sheng Yu^1^, Yaosheng Lu^1,2^, Shun Long^1^, Huijin Wang^1^, Ruiyu Qiu^1^, Zhanhong Ou^1^, Minghong Zhou^1^, Dengjiang Zhi^1^, Mengqiang Zhou^1^, Xiaosong Jiang^1^, and Gaowen Chen^3^*

^1^College of Information Science and Technology, Jinan University, Guangzhou 510632, China;

^2^Guangdong Provincial Key Laboratory of Traditional Chinese Medicine Information Technology, Jinan University, Guangzhou 510632, China;

^3^Obstetrics and Gynecology Center, Zhujiang Hospital, Southern Medical University, Guangzhou 510280, China.

Correspondence and requests for materials should be addressed to Jieyun Bai ([baijieyun@jnu.edu.cn](mailto:baijieyun@jnu.edu.cn) or [bai_jieyun@126.com](mailto:bai_jieyun@126.com)) or Gaowen Chen ([cgw2012@163. com](mailto:cgw2012@163.com))

**Supplemental Table 1.** The configurations of the DBSN.

| Layer | Input | Operator | Output |
| --- | --- | --- | --- |
| *F_1_* | Original image | (Conv+GN+ReLu)×2 | En_Map1-0  (512×384×32) |
|  | En_Map1-0 | 2×2 Max Pooling | En_Map1-1  (256×192×32) |
| *F_2_* | En_Map1-1 | (Conv+GN+ReLu)×2 | En_Map2-0  (256×192×64) |
|  | En_Map2-0 | 2×2 Max Pooling | En_Map2-1  (128×96×64) |
| *F_3_* | En_Map2-1 | (Conv+GN+ReLu)×2 | En_Map3-0  (128×96×128) |
|  | En_Map3-0 | 2×2 Max Pooling | En_Map3-1  (64×48×128) |
| *F_4_* | En_Map3-1 | (Conv+GN+ReLu)×2 | En_Map4-0  (64×48×256) |
|  | En_Map4-0 | 2×2 Max Pooling | En_Map4-1  (32×24×256) |
| *F_5_* | En_Map4-1 | (Conv+GN+ReLu)×2 | En_Map5-1  (32×24×512) |
| *L_1_* | En_Map5-1 | (Conv+GN+ReLu)×2 | De_L_Map1-1  (32×24×256) |
| *L_2_* | De_L_Map1-1 | (Conv+GN+ReLu)×2 | De_L_Map2-1  (32×24×128) |
| *L_3_* | De_L_Map2-1 | (Conv+GN+ReLu)×2 | De_L_Map3-1  (32×24×64) |
| *L_4_* | De_L_Map3-1 | (Conv+GN+ReLu)×2 | De_L_Map4-1  (32×24×32) |
| *D_L_* | De_L_Map4-1 | 1×1Conv + Softmax | Lower_Output:  (32×24×3) |
| *U_1_* | De_L_Map1-1 | Up-sampling+  Conv+GN+ ReLu | De_L_U1-1  (64×48×256) |
| *U_2_* | En_Map4-0  De_L_U1-1 | (Conv+GN+ReLu)×2 | De_U_Map2-1  (64×48×128) |
|  | De_L_Map2-1 | Up-sampling+  Conv+GN+ ReLu | De_L_U2-1  (128×96×128) |
|  | De_U_Map2-1 | Up-sampling+  DC+GN+ ReLu | De_U_U2-1  (128×96×128) |
|  | De_L_U2-1  De_U_U2-1 | Attention Gate | De_L_U AG2-1  (128×96×128) |
| *U_3_* | En_Map3-0  De_U_U2-1  De_L_U AG2-1 | (Conv+GN+ReLu)×2 | De_U_Map3-0  (128×96×64) |
|  | De_L_Map3-1 | Up-sampling+  Conv+GN+ ReLu | De_L_U3-1  256×192×64 |
|  | De_U_Map3-0 | Up-sampling+  DC+GN+Relu | De_U_U3-1  (256×192×64) |
|  | De_L_U3-1  De_U_U3-1 | Attention Gate | De_L_U AG3-1  (256×192×64) |
|  | En_Map2-0  De_U_U3-1  De_L_U AG3-1 | (Conv+GN+ReLu)×2 | De_U_Map3-1  (256×192×64) |
| *U_4_* | De_L_Map4-1 | Up-sampling+  Conv+GN+ReLu | De_L_U4-1  (512×384×32) |
|  | De_U_Map3-1 | Up-sampling+  DC+GN+Relu | De_U_U4-1  (512×384×32) |
|  | De_L_U4-1  De_U_U4-1 | Attention Gate | De_L_U AG4-1  (512×384×32) |
|  | En_Map1-0  De_U_U4-1  De_L_U AG4-1 | (Conv+GN+ReLu)×2 | De_U_Map4-1  512×384×32 |
| *D_U_* | De_U_Map4-1 | 1×1Conv + Softmax | Upper_Output:  (512×384×3) |

Note: Conv: Convolution operator; DC: Deformable Convolution operator; GN: Group normalization.

**Supplemental Table 2.** Performance of the network using different weights (***w_L_***) on Acc, ***Dice_all_***, ***Dice_PS_*** and ***Dice_FH_*** on our private dataset. (The blacked part denoted those indicators are the best.)

| ***w_L_*** | ***Acc*** | ***Dice_all_*** | ***Dice_PS_*** | ***Dice_FH_*** |
| --- | --- | --- | --- | --- |
| 0.1 | 0.9865 | 0.9291 | 0.9005 | 0.9327 |
| **0.2** | **0.9874** | **0.9338** | **0.9101** | **0.9366** |
| 0.3 | 0.9867 | 0.9301 | 0.9096 | 0.9326 |
| 0.5 | 0.9868 | 0.9310 | 0.9059 | 0.9341 |
| 1.0 | 0.9867 | 0.9298 | 0.9040 | 0.9330 |

Note: ***w_L_*** denotes the weight of the loss function for the lower branch of DBSN.

**Supplemental Table 3.** Performance of different networks on Acc, ***Dice_all_***, ***Dice_PS_*** and ***Dice_FH_*** on our private dataset. (The blacked part denoted those indicators are the best.)

| ***Model*** | ***Acc*** | ***Dice_all_*** | ***Dice_PS_*** | ***Dice_FH_*** |
| --- | --- | --- | --- | --- |
| *DBSN* | **0.9874** | 0.9338 | 0.9101 | 0.9366 |
| *DBSN-AG* | **0.9874** | **0.9339** | 0.9084 | **0.9369** |
| *DBSN-LB* | 0.9873 | 0.9334 | **0.9156** | 0.9355 |
| *DBSN-DC* | 0.9864 | 0.9292 | 0.9083 | 0.9317 |

Note: DBSN, DBSN-AG, DBSN-LB and DBSN-DC denotes, Double Branch Segmentation Network (DBSN), DBSN without the attention gate (AG), DBSN without the low branch (LB) and DBSN without deformable convolutional blocks (DC), respectively.

**Supplemental Table 4.** Performance of different networks on ***D_U_***, ***D_L_*** and ***A_X_*** on our private dataset. (The blacked part denoted those indicators are the best.)

| ***Model*** | ***ED_U_* (mm)** | ***ED_L_* (mm)** | ***A_X_* (**°**)** |
| --- | --- | --- | --- |
| *DBSN* | **4.21** | 3.62 | 4.15 |
| *DBSN-AG* | 4.51 | 3.67 | 4.48 |
| *DBSN-LB* | 4.47 | **3.52** | **3.97** |
| *DBSN-DC* | 4.69 | 3.84 | 4.30 |

Note: DBSN, DBSN-AG, DBSN-LB and DBSN-DC denotes, Double Branch Segmentation Network (DBSN), DBSN without the attention gate (AG), DBSN without the low branch (LB) and DBSN without deformable convolutional blocks (DC), respectively.

**Supplemental Table 5.** Performance of different networks on $\boldsymbol{\Delta}$*AoP_Mean*, $\boldsymbol{\Delta}$*AoP_Median* and $\boldsymbol{\Delta}$*AoP_Std* on our private dataset. (The blacked part denoted those indicators are the best.)

| ***Model*** | $\boldsymbol{\Delta}$***AoP_Mean* (°)** | $\boldsymbol{\Delta}$***AoP_Median* (°)** | $\boldsymbol{\Delta}$***AoP_Std* (°)** |
| --- | --- | --- | --- |
| *DBSN* | **5.993** | 5.851 | **3.872** |
| *DBSN-AG* | 6.490 | 5.690 | 5.151 |
| *DBSN-LB* | 6.200 | **5.005** | 5.096 |
| *DBSN-DC* | 6.456 | 5.180 | 6.043 |

Note: DBSN, DBSN-AG, DBSN-LB and DBSN-DC denotes, Double Branch Segmentation Network (DBSN), DBSN without the attention gate (AG), DBSN without the low branch (LB) and DBSN without deformable convolutional blocks (DC), respectively.

**Supplemental Table 6.** Performance of different networks on $\boldsymbol{\Delta}$*AoP_Mean*, $\boldsymbol{\Delta}$*AoP_Median* and $\boldsymbol{\Delta}$*AoP_Std* on the public JNU-IFM dataset. (The blacked part denoted those indicators are the best.)

| ***Model*** | ***Dice_all_*** | ***Dice_PS_*** | ***Dice_FH_*** |
| --- | --- | --- | --- |
| *DBSN* | 0.9180 | 0.8726 | 0.9234 |
| *DBSN-AG* | **0.9198** | 0.8740 | **0.9253** |
| *DBSN-LB* | 0.9188 | **0.8760** | 0.9240 |
| *DBSN-DC* | 0.9152 | 0.8696 | 0.9208 |

**Supplemental Table 7.** Performance of different networks on $\boldsymbol{\Delta}$***AoP_Mean***, $\boldsymbol{\Delta}$***AoP_Median*** and $\boldsymbol{\Delta}$***AoP_Std*** on the public JNU-IFM dataset. (The blacked part denoted those indicators are the best.)

| ***Model*** | $\boldsymbol{\Delta}$***AoP_Mean* (°)** | $\boldsymbol{\Delta}$***AoP_Median* (°)** | $\boldsymbol{\Delta}$***AoP_Std* (°)** |
| --- | --- | --- | --- |
| *DBSN* | 5.178 | 4.229 | 4.579 |
| *DBSN-AG* | 5.188 | 4.260 | 4.542 |
| *DBSN-LB* | 5.133 | 4.211 | **4.338** |
| *DBSN-DC* | **5.110** | **4.181** | 4.607 |

**Supplemental Table 8.** Performance of U-net and DBSN on our private dataset.

| ***Model*** | ***ACC*** | ***Dice_all_*** | ***Dice_PS_*** | ***Dice_FH_*** | ***ASD*** | $\boldsymbol{\Delta}$***AoP_Mean* (°)** |
| --- | --- | --- | --- | --- | --- | --- |
| U-net | 0.984 | 0.913 | 0.889 | 0.916 | 10.989 | 8.00 |
| **DBSN** | **0.987** | **0.934** | **0.910** | **0.937** | **6.268** | **5.993** |


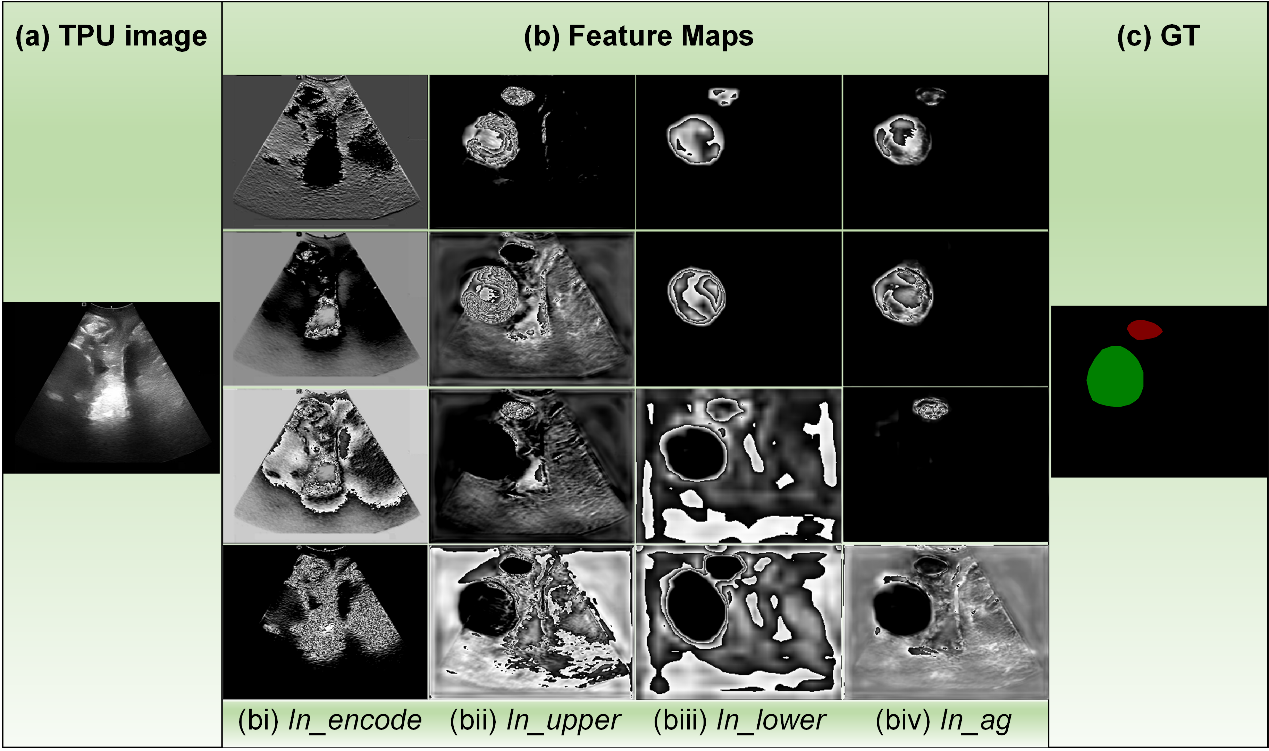


**Supplemental Fig. 1**. Example feature maps produced by the DBSN. **(a)** The input original image. **(b)** Four feature maps produced by the layer with 32 channels of each branch. **(bi)** Feature maps of the encoder branch (*In_encoder*); **(bii)** Feature maps of the upper decoder branch (*In_upper*); **(biii)** Feature maps of the lower decoder branch (*In_lower*); **(biv)** Feature maps of the attention gate (*In_ag*); **(c)** The corresponding ground truth (GT).


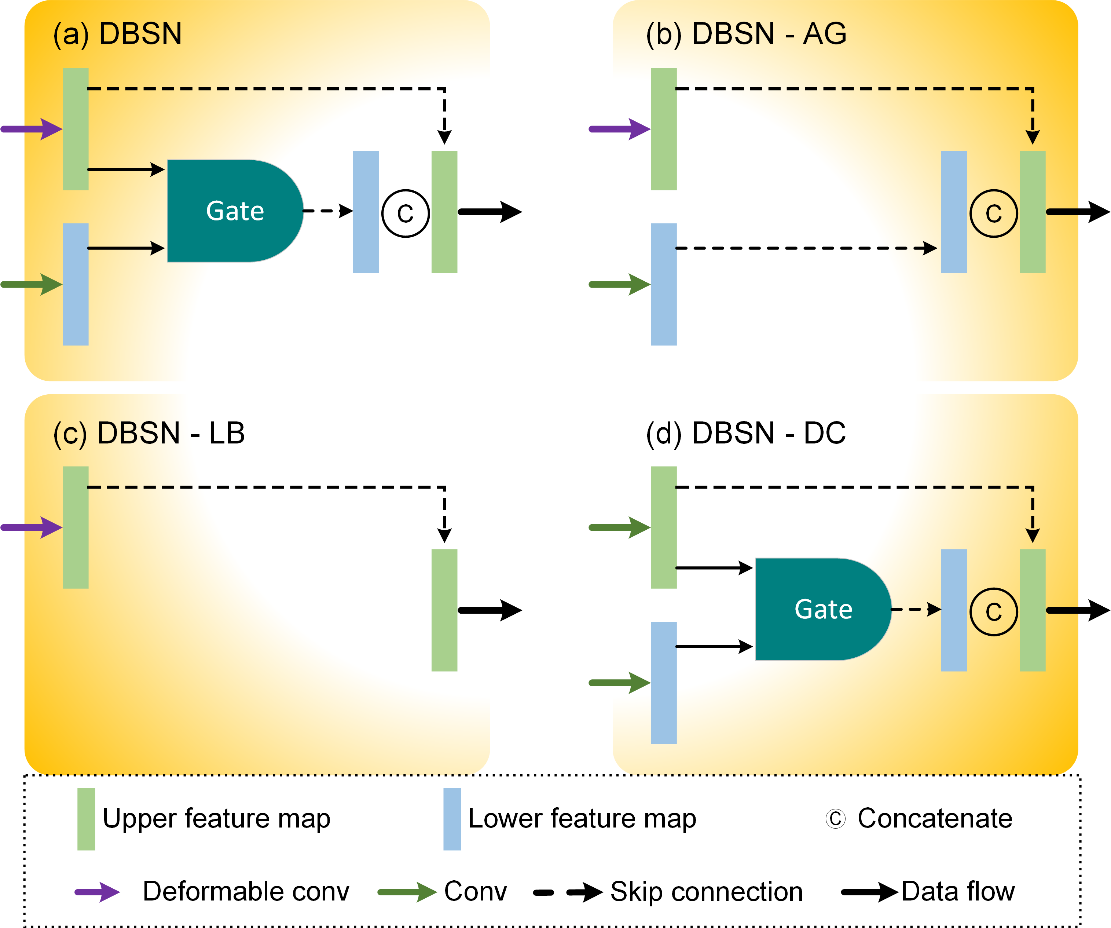


**Supplemental Fig. 2**. Four different architectures for the ablation study. **(a)** Our Double Branch Segmentation Network (DBSN) model contains the lower branch (LB), attention gate (AG) and deformable convolution (DC). **(b)** DBSN without AG. **(c)** DBSN without LB. **(d)** DBSN without DC. Note: the purple line indicates Up-sampling + 3$\times$3 DC + Group-normalization + ReLU, whereas the green line indicates Up-sampling + 3$\times$3 convolution + Group-normalization + ReLU. The green and blue rectangular blocks respectively represent feature maps of the upper branch and LB.
